# Enhanced Dielectric Permittivity of Optimized Surface Modified of Barium Titanate Nanocomposites

**DOI:** 10.3390/polym12040827

**Published:** 2020-04-05

**Authors:** Udhay Sundar, Zichen Lao, Kimberly Cook-Chennault

**Affiliations:** 1 Portland Technology Development, Intel Corporation, Portland, OR 97124, USA; udhay.sundar@gmail.com; 2 Mechanical Engineering and Applied Mechanics, University of Pennsylvania, Philadelphia, PA 19104, USA; lao0910@seas.upenn.edu; 3 Mechanical and Aerospace Engineering, Rutgers, the State University of New Jersey, Piscataway, NJ 08854, USA

**Keywords:** dielectric, capacitor, embedded energy storage, permittivity

## Abstract

High permittivity polymer-ceramic nanocomposite dielectric films take advantage of the ease of flexibility in processing of polymers and the functionality of electroactive ceramic fillers. Hence, films like these may be applied to embedded energy storage devices for printed circuit electrical boards. However, the incompatibility of the hydrophilic ceramic filler and hydrophobic epoxy limit the filler concentration and therefore, dielectric permittivity of these materials. Traditionally, surfactants and core-shell processing of ceramic fillers are used to achieve electrostatic and steric stabilization for adequate ceramic particle distribution but, questions regarding these processes still remain. The purpose of this work is to understand the role of surfactant concentration ceramic particle surface morphology, and composite dielectric permittivity and conductivity. A comprehensive study of barium titanate-based epoxy nanocomposites was performed. Ethanol and 3-glycidyloxypropyltrimethoxysilan surface treatments were performed, where the best reduction in particle agglomeration, highest value of permittivity and the lowest value of loss were observed. The results demonstrate that optimization of coupling agent may lead to superior permittivity values and diminished losses that are ~2–3 times that of composites with non-optimized and traditional surfactant treatments.

## 1. Introduction

**Figure 1:**
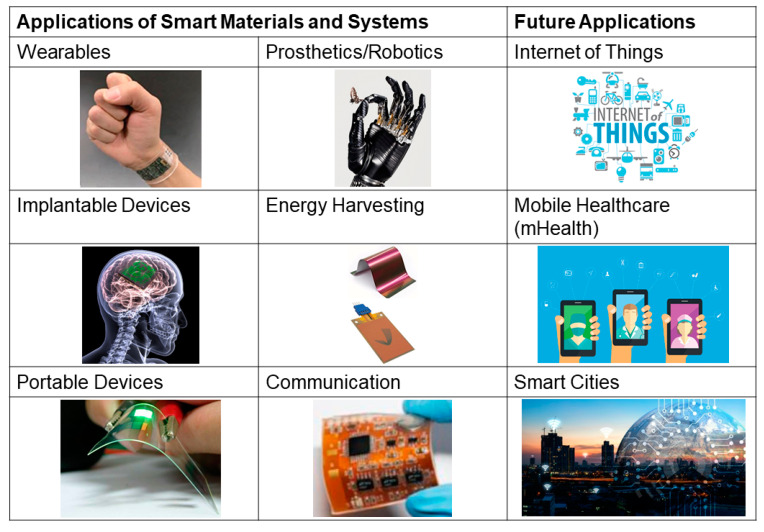
Overview of uses and applications of the proposed materials.

Dielectric materials are ubiquitously used today and novel materials with extraordinary properties are being explored for advanced applications, such as electric stress control for cables and joints to shield current loss at concentrated points, energy storage as capacitors [1,2,3], embedded passive devices, photovoltaics and battery electrode materials that enhance the ionic conductivity and thermal stability of the electrolyte as shown in Figure 1. Typically, materials used in these applications must possess high dielectric and mechanical strength, high thermal conductivity to alleviate thermal stresses, good ductility, and high glass transition and curie temperatures to prevent premature failure.

Either ceramic dielectric oxides or polymers are used for majority of the aforesaid applications because ceramic oxides possess high dielectric permittivity (ε_r_ = 200–10,000 [4,5,6,7]) and high stiffness (elastic modulus between 50–100 GPa [8,9,10,11]). These materials also suffer from high dielectric loss over broad frequency bands and relatively high mechanical stiffness, which makes them susceptible to premature failure when subjected to extensive cyclic operation and inherently low breakdown field strength. These characteristics limits their available energy density for many operations such as energy storage and IOT applications. Polymer-based dielectric materials, on the other hand, have high breakdown field strengths, low dielectric losses and are inherently ductile. These inherent properties make them easy to process into various shapes and films and easier to 3D print. Nevertheless, these materials have very low dielectric permittivity and lower mechanical stiffness.

Embedded capacitor technology is an important technique for miniaturization and production of high performance of electronic packaging systems. High dielectric constant (ε_r_) polymer-ceramic composites have been of great interest as embedded capacitor materials because of their ease of fabrication, compatibility with printed wiring board (PWB) technology, and high relative permittivity (5–20,000) [12,13,14,15]. In order to design more effective capacitors, it is essential to achieve high permittivity of the composite. Many workers have demonstrated that the dielectric constants of polymer-ceramic composites could be enhanced by adding conductive particles in insulative polymeric matrix as a third phase in the composite [16,17,18,19,20]. This enhances the overall permittivity of the composite by forming conductive networks. However, these composites suffer from high dielectric losses due to increased dielectric loss and low breakdown strengths, which makes them less amenable to electrical polarization [21,22,23].

In general, composites that contain high filler loadings (vol.% > 50) exhibit high overall permittivity values. Nanoparticles in polymer matrices have several advantages over homogenous ceramic dielectric materials. Some of these advantages include enhanced dielectric breakdown strength and improved voltage endurance over the life of the sample [24,25]. A minor stumbling block is the proclivity of nanoparticles to agglomerate by forming aggregates within the matrix [26,27]. This agglomeration is attributed to two main factors: high surface area to volume ratios and van der Waals forces that arise between all molecules and particles. High surface area to volume rations causes nanoceramic particles to form bonds with one another, in order to diminish the surface energy. The second factor, van der Waals forces, arise between all molecules and particles. Nanoparticles of metals, metal oxides and ceramic materials that are not coated have robust van der Waals forces [26,27]. Inter-particle agglomeration dues to surface energy can be reduced by using surfactants and coupling agents that form covalent bonds with the nanoparticles.

Moreover, the incompatibility of inorganic-organic constituent materials in the composite exacerbate difficulties in processing of these films [13,14,28,29,30,31]. The surface of BaTiO_3_ particles in BaTiO_3_/epoxy composites have residual hydroxyl groups, which are hydrophilic in nature. On the other hand, the epoxy resin and organic solvent used in the composite are hydrophobic. Therefore, BaTiO_3_ tends to agglomerate and separate from the organic solvent or resin, which causes processing difficulties [13,14,32]. Hence, dielectric polymer nanocomposites that have high-volume fractions of ceramic filler have been of interest due to obstacles associated with processing and particle dispersion.

Identification parameters pertaining to the surface modification of ceramics could address both of these problems. For example, researchers have looked at modifying BaTiO_3_ by treating it with hydrogen peroxide to hydroxylate the surface [33,34,35,36,37]. The compatibility between the inorganic fillers and polymer materials can be enhanced with the use of surface modifiers such as macromolecular surface modifiers [38,39] and small-molecular weight surface active agents, such as, silane and titanate coupling agents [40,41,42,43]. Coupling agents such as these have two functional groups, wherein one reacts with either BaTiO_3_ and the other with the epoxy by forming covalent bonds. Silane coupling agents were studied in dielectric polymer composites such as epoxy/aluminum [44] and epoxy/fosterite (Mg_2_SiO_4_) [45].

Zhou et al. [44] surface treated aluminum filler with coupling agents (silane KH550, silane KH560, titanate NXT-102, titanate NXT-201) prior to embedding the filler into a diglycidyl ether of bisphenol A-type epoxy resin, and examined the aluminum filler microstructure and composite dielectric properties. In this study, the concentration of Al filler ranged from 0 to 70% by weight. It was found that the surface treatment of the aluminum, rendered enhanced dielectric constants, e.g., 34 juxtapose 19.6 for treated and pristine filler aluminum powder at 70% weight concentration, respectively and reduced dielectric loss values. It was concluded that the use of coupling agents improved the interfacial bonding strength between the aluminum and the epoxy resin and decreased the voids and defects at the phase interfaces. These workers also indicated that the KH-560 silane coupling agent had an epoxide as one of its end groups, and three hydroxyls at its other end after reacting with the Al particles. Others such as Sasikala et al. [45] investigated the influence of silane coupling agents on the microstructure, dielectric and thermal properties of epoxy-forsterite (Mg_2_SiO_4_) composites. In this work, it the dielectric permittivity was found to increase with the composite fabricated using silane coupling agent due to uniform dispersion of the filler within the matrix leading to enhanced polarization from the increased dipole-dipole interaction [46]. In this work, the dielectric permittivity was found to be ~3.7 and ~3.9 at 7 GHz for untreated and surface treated fillers, respectively. Similarly, Iijima et al. [21] modified the surface of BaTiO_3_ particles using by a combination of 3-glycidoxypropyltrimethoxysilane (GPTMS) and a variety of solvents, where the goal was to examine the effects the GPTMS and solvents on composite performance. In this work, the particles were modified in water, ethanol and xylene, along with GPTMS. At 1 kHz the dielectric constant for BaTiO_3_ nanoparticles that were untreated was 37 and those treated in water, ethanol and xylene were 31, 30 and 52 respectively. They concluded that the composites that were surface modified in xylene showed the most improved properties, reduced surface roughness and less voids compared with the non-surface treated composites. Huang et al. [47] investigated the influence of the surface modified BaTiO_3_ nanoparticles on the electrical, thermophysical and micromechanical properties of ethylene-vinyl acetate (EVM) vulcanizates. The nanoparticles were surface modified using gamma-aminopropyltriethoxysilane. The workers found that even at high concentrations (~50% volume fraction) of BaTiO_3_, the filler powder uniformly dispersed within the matrix material, and attributed this to the substitution of hydroxyl groups on the BaTiO_3_ nanoparticles surface. They surmised that this process led to two effects: lowering of the interfacial tension between the separated phases and prevention of coalescence of the nanoparticles during processing. The dielectric constant of 50% BT-EVM was 15 at 1 kHz. It was reported that the incorporation of surface modified BaTiO_3_ nanoparticles into the EVM matrix increased the permittivity, thermal conductivity (from 0.295 Wm^−1^ K^−1^ for pure EVM matrix to 0.87 Wm^−1^ K^−1^ for EVM-BaTiO_3_ (0.5)). Similar to Iijima et al. [21], they attributed the higher dielectric strength of the nanocomposites at high BaTiO_3_ loading levels to the good dispersion state of BaTiO_3_ particles in EVM matrix and excellent interfacial adhesion between the polymer matrix and BaTiO_3_ nanoparticles [48,49]. Zeng et al. [50] fabricated bismaleimide-triazine resin/barium titanate (BT/ BaTiO_3_) nanocomposite films, via a mix and cast approach, where the nanoparticles were surface treated using gamma-glycidoxypropyl trimethoxysilane (KH-560). It was reported that for the composite with 70 wt.% of BaTiO_3_, the effective dielectric constant at room temperature reached 23.63, and with a dielectric loss of 0.0212 at 100 Hz. The dielectric properties of the nanocomposite films were nearly frequency-independent, which was attributed to the excellent dispersion of BaTiO_3_ nanoparticles in the BT matrix. The surface modification of BaTiO_3_ nanoparticles with silane coupling agent resulted in excellent dispersion and enhanced the interaction between BaTiO_3_ and the BT matrix.

Dalle Vacche et al. [51] investigated the influence of varying size BaTiO_3_ particles which were surface modified with aminopropyl triethoxy silane, and incorporated into poly (vinylidene fluoride–trifluoroethylene) P(VDF-TrFE) up to 60% volume fraction. Three BaTiO_3_ powders were used < 2 µm (average particle size 1.1 µm), 0.7 µm and 0.2 µm. The dielectric permittivity for the composites containing 60% volume fraction of BaTiO_3_ was ~113 (1.1 µm) for surface treated and ~100 for non-surface treated composites and, ~100 (0.7 µm) for the surface treated samples and ~80 for the untreated samples and ~85 (0.2 µm) for the surface treated samples and ~75 for the untreated samples at 1 kHz, respectively. The dissipation factor was recorded to be ~0.1 for the untreated sample and ~0.05 for the surface treated samples. Surface modification was found to be effective for improving the dispersion of the ceramic in the matrix therefore reducing particle aggregates and enhancing the compatibility and adhesion of the P (VDF–TrFE) matrix to the inorganic filler. The composites also showed lower dielectric losses. Some workers have also illustrated a method for using point defects to explore the relationships between conduction loss/polarization and dielectric behaviour in semiconductor and graphitized carbon. [52]

In order to understand the role of interface in highly filled polymer nanocomposites due to a current lack of comprehensive research work Huang et al. [24] conducted the surface modification of BaTiO_3_ with 6 different types of surface chemistry was carried out. Epoxy resin (DGEBA) was used as the overlaying polymer matrix. Silane coupling agents (3-mercaptopropyltrimethoxysilane (KBM 803), 3-aminopropyltrimethoxysilane (KBM 903) and 2-(3,4-epoxycyclohexyl) ethyltrimethoxysilane (KBM 303) were used to surface modify BaTiO_3_ nanoparticles. All the nanocomposites contain 50 vol % BaTiO_3_ nanoparticles. The dielectric permittivity of pristine BaTiO_3,_ BaTiO_3_ treated with H_2_O_2,_ BaTiO_3_ treated with KBM 803, BaTiO_3_ treated with KBM 903 and BaTiO_3_ treated with KBM 303 were found to be 30, 31, 25, 28 and 28 respectively at 1 kHz. They also reported that surface modification of BaTiO_3_ with silane coupling agents resulted in decreased dielectric loss tangent and weak dielectric dispersion. The majority of the existing literature does not provide insight regarding the influence of concentration of the coupling agent as a function of permittivity, both real and imaginary. As a result, there is a limited understanding on how the variability of the concentration of the coupling agent impacts the properties of the dielectric composite. This work aimed to understand how concentration of the coupling agent influences these parameters to develop an effective dielectric composite wherein, BaTiO_3_/epoxy thick film composites were fabricated (volume fractions of BaTiO_3_ were varied from 0.10 to 0.60). Nano-sized barium titanate (BaTiO_3_) filler was surface modified using ethanol (3 mL per unit gram of BaTiO_3_) and varying concentrations (in terms of volume fraction of the combined mixture) of silane coupling agent (0.01, 0.015, 0.020 and 0.025).

## 2. Materials and Methods

### 2.1. Materials

The materials used in this work were BaTiO_3_ (Sigma-Aldrich, Saint louis, MO, USA, <100 nm particle size, ≥99%) in the cubic crystalline phase, Epofix Cold-Setting embedding resin (Bisphenol-A-Diglycidylether, Electron Microscopy Sciences, Hatfield, PA, USA) [53], Triethylenetetramine (epoxy hardener) and 3-Glycidyloxypropyltrimethoxysilane (Sigma-Aldrich, Saint louis, MO, USA, ≥98%, KH-560). The dielectric and physical properties of the constituent materials: BaTiO_3_, DGEBA epoxy and KH-560 are presented in Table 1 and Table 2, respectively.

### 2.2. Experimental Method

#### 2.2.1. Surface Modification of BTO with Ethanol

The surface modification of the BaTiO_3_ (BTO) nano-powder using ethanol was used as the baseline datum for all studies, where it was used as a dispersant to aid in breaking down the particle-particle agglomerations [20]. The method for surface of modification using the ethanol and time study detailing the strategy for ascertaining agglomeration and dispersion of BTO using a Zeiss Sigma Field Emission scanning electron microscope (SEM, Oberkochen, Germany) and an Oxford INCA PentaFET x3 8100 energy dispersive X-ray spectroscopy (EDS, Oberkochen, Germany) is described in [55].

#### 2.2.2. Surface Modification of BTO with Silane Coupling Agent

*Glycidyloxypropyl* type silane was chosen as the organic functional group in this work because glycidylfunctional epoxy resin i.e., dyglycidyl ether of bisphenol A (DGEBA) is the matrix material for the nanocomposites [50,56], this coupling agent is suitable for this type of matrix material. Four different volume fractions of coupling agent were used in this study: 0.01, 0.015, 0.020 and 0.025 (*v/v* i.e., volume fraction percentage) to surface modify the BaTiO_3_ nano-powder using a process described by [55]. Subsequent sol gel synthesis and spin coat and film deposition processes were performed using the process describe in [55].

#### 2.2.3. Film Characterization

The capacitance, resistance and conductance were measured using a Hewlett Packard 4194A Impedance/Gain-Phase Analyzer (Keysight Technologies, Santa Rosa, CA, USA) over a frequency between 2000 Hz and 40 MHz. This large frequency sweep provides insight into the electromechanical behavior of the sample with increasing frequency. The complex dielectric permittivity consists of the real and imaginary part. The real part of the complex permittivity is known as the dielectric constant and it is a measure of the charge storage abilities of the material. The imaginary part is the loss component i.e., dielectric loss or dissipation. This loss could originate from heat or from ohmic conduction of the material. The dielectric constant was calculated using Equation (1).

(1) $$\varepsilon_{r}=\frac{Ct}{A\varepsilon_{0}}$$

In Equation (1), *C, A,* ε_0_ and *t* are the capacitance in Farads, surface area of the sample, permittivity of free space ~8.854 × 10^−12^ F m^−1^, and thickness of the sample, respectively. The impedance, *Z*, was measured using a HP 4194A impedance analyzer. The real and imaginary parts of impedance, are resistance, *R* and reactance, *X*, respectively as expressed in Equation (2).

(2) Z=R+jX

The real part of impedance, i.e., resistance was then used to calculate resistivity, so as to normalize it for units of length as can be seen in Equation (3).

(3) $$\rho =\frac{RA}{t}$$

Similarly, the admittance, *Y*, is also measured by the HP 4194A impedance analyzer which, like impedance, is also a complex quantity as shown in Equation (4).

(4) Y=G+jB

where *G* is conductance and *B* is susceptance. Conductivity is calculated using Equation (5).

(5) $$\sigma =\frac{Gt}{A}$$

The effective permittivity of a polymer nanocomposite can be calculated by knowing the individual permittivity of the ceramic fillers and polymer matrix along with filler loading level i.e., volume fraction of the filler in the polymer matrix. There are several models that can be used to determine the effective permittivity of the composite.

The experimental values for the dielectric constant are compared to four theoretical models: (1) the Maxell-Garnett Equation, (2) the Bruggeman Self-Consistent Effective Medium Approximation, (3) the Lichtenker’s Formula and (4) Jaysundere-Smith Equation. The Maxwell-Garnett model is typically applicable to composites composed of continuous media filled with spherical particles [57,58,59], where the polymer matrix is assumed as an isotropic medium with a dielectric permittivity $\varepsilon_{m}$ and the particles are assumed to be in the shape of spheroids with permittivity vales equal to $\varepsilon_{f}$. The volume fraction of filler particles,$\;\emptyset_{f}$, and the resulting volume fraction of the matrix is expressed as $\emptyset_{m}=1-\emptyset_{f}$. The aforementioned expression assumes the filler and matrix components have no dielectric loss and that the distance between the inclusions are greater than their characteristic sizes according to [60,61,62,63,64]. This model expressed in Equation (6) is not limited by the resistivity of the filler or the polymer matrix.

(6) $$\varepsilon_{eff}=\varepsilon_{m}\left[1+\frac{3\emptyset_{f}\left(\varepsilon_{f}-\varepsilon_{m}\right)}{\emptyset_{m}\left(\varepsilon_{f}-\varepsilon_{m}\right)+3\varepsilon_{m}}\right]$$

The Bruggeman Self-Consistent Effective Medium Approximation model is more applicable for composites that contain slightly larger filler concentrations in comparison to the Maxwell-Garnett equation (which is more effective for lower filler loadings). This model also yields better results when the fillers particles are very close to one another and even agglomerate [57,64]. The formula of this model for spherical fillers is expressed in Equation (7).

(7) $$\left(1-\emptyset_{f}\right)\frac{\varepsilon_{m}-\varepsilon_{eff}}{\varepsilon_{m}+2\varepsilon_{eff}}+\emptyset_{f}\frac{\varepsilon_{m}-\varepsilon_{eff}}{\varepsilon_{m}+2\varepsilon_{eff}}=0.$$

This can be further solved for the final Bruggeman’s formula [65,66], which is shown as Equation (8).

(8) εf−εeffεeff13=(1−∅f)(εf−εm)εm13

This equation is expected to hold for $\emptyset_{f}$ values up to 0.5, with the constraint that the dispersed spheres do not form a percolative path throughout the medium. On the other hand, the Lichtenker formula is a logarithmic mixture formula and is effective in calculating the permittivity of the polymer composite. The Lichtenecker’s formula is shown in Equation (9) [67,68,69].

(9) $$\varepsilon_{eff}^{\alpha }=\emptyset_{m}\varepsilon_{m}^{\alpha }+\emptyset_{f}\varepsilon_{f}^{\alpha }$$

In Equation (9), $\varepsilon_{eff}$ is the effective permittivity of the composite, and $\varepsilon_{m}$ and $\varepsilon_{f}$ are the permittivity values of the polymer matrix and ceramic filler, and $\emptyset_{f}$ and $\emptyset_{m}$ are the volume fraction of fillers and matrix, respectively. The parameter α varies from −1 to +1 and is considered to describe the transition from anisotropy (α = −1) to isotropy (α = +1) [57]. The Maxwell-Garnett equation is typically only valid for lower concentration of fillers since the interaction between filler particles is relatively weak, and at higher volume fractions the interaction between the fillers becomes significant because the distance between them reduces [57,64]. Jayasundere and Smith [70] proposed a more realistic mixing rule. They calculated the electric field with a dielectric sphere embedded in a continuous dielectric medium by taking into account polarization of adjacent particles and arrived at Equation (10).

(10) $$\varepsilon_{eff}=\frac{\emptyset_{m}\varepsilon_{m}+\emptyset_{f}\varepsilon_{f}\frac{3\varepsilon_{m}}{2\varepsilon_{m}+\varepsilon_{f}}\left[1+3\emptyset_{f}\frac{\left(\varepsilon_{f}-\varepsilon_{m}\right)}{2\varepsilon_{m}+\varepsilon_{f}}\right]}{\emptyset_{m}+\emptyset_{f}\frac{3\varepsilon_{m}}{2\varepsilon_{m}+\varepsilon_{f}}\left[1+3\emptyset_{f}\frac{\left(\varepsilon_{f}-\varepsilon_{m}\right)}{2\varepsilon_{m}+\varepsilon_{f}}\right]}$$

## 3. Results and Discussion

The Results and Discussion Section is divided into two subsections. The first subsection describes the dielectric permittivity of ethanol- and silane-surface treated samples, while the second subsection focuses on the conductivity samples.

### Dielectric Permittivity

#### Dielectric Permittivity (BTO-Ethanol)

**Figure 2:**
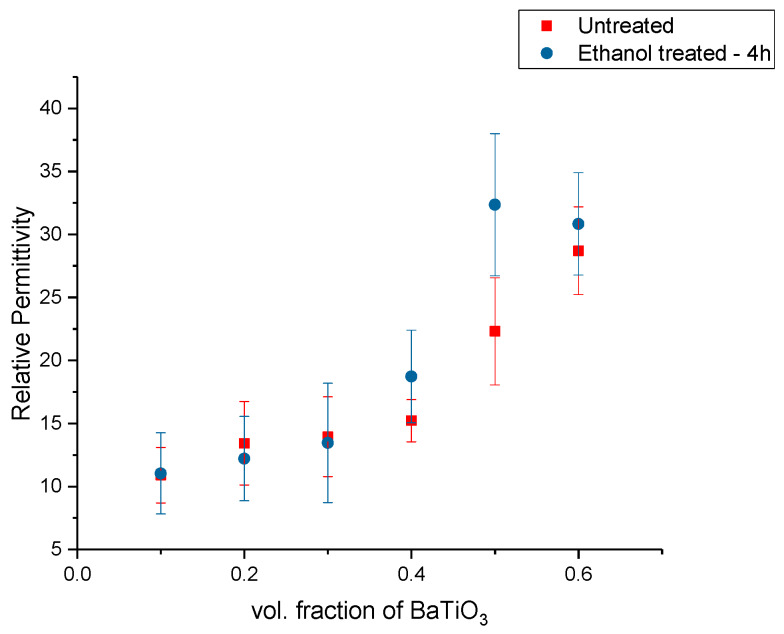
The relative permittivity of the BaTiO_3_-epoxy composite fabricated using BaTiO_3_ nanoparticles that were surface modified using ethanol for 4 h is plotted as a function of vol. fraction. The measurements were taken at 20 MHz.

The BaTiO_3_-epoxy composites were fabricated using BaTiO_3_ nanoparticles that were surface modified using ethanol for 4 h. The relative permittivity or dielectric constant was measured at 20 MHz and plotted as function of increasing volume fraction of the nanoparticle fillers, as shown in Figure 2. In Figure 2, the relative permittivity is plotted as a function of volume fraction. In this figure, the permittivity increases as a function of volume fraction of the BaTiO_3_ filler. The ethanol is a dispersant in the composites, where it improves the dispersion of the filler. However, it does not act as a binding or molecular bridge between fillers and polymers [71,72,73,74].

#### Dielectric Permittivity (BTO-Silane Coupling Agent)

To observe the influence of the concentration of coupling agent on BTO nanoclusters, permittivity and dissipation spectra were obtained. The BaTiO_3_ nanoparticles were then surface modified using silane coupling agent, KH-560 at a volume fraction of 0.01, 0.015, 0.020 and 0.025. The coupling agent enables more interfacial regions between BaTiO_3_ and epoxy creating a more uniform and homogeneous dispersion of nanoparticles within the matrix. The volume fraction of BaTiO_3_ for these composites was also varied from 0.1 through 0.6. By varying the concentration of the coupling agent during surface treatment it enables us to gain a broader understanding of its interaction with BaTiO_3_ nanoparticles. The dielectric permittivity was measured using a HP 4194A impedance analyzer. These measurements were taken at a broad frequency range between 2000 Hz and 40 MHz. This enabled us to observe the behavior of the permittivity values with increasing frequency and to understand the effect or lack thereof of different polarization mechanisms. Here too, the BaTiO_3_ volume fraction was varied from 0.1 to 0.6 for each concentration of surface modification. All the composites were corona poled at 30 kV/mm to ensure consistency of data. Figure 2 shows the real part of permittivity of the untreated or pristine BaTiO_3_/epoxy composites plotted against increasing frequency. As expected, one can see that the permittivity of the entire composite increases with increasing volume fraction of BaTiO_3_.

The frequency dependence of permittivity shows a decrease in permittivity with increasing frequency for composites and resins [75]. The dielectric relaxations depicted in Figure 2, Figure 6, Figure 9, Figure 12 and Figure 17 can be explained by polarization mode changes. The polarization of dielectrics in alternating current fields has mode changes as frequency changes. Initially, at low frequencies, the total polarization manifests itself completely [12,76]. However, at high frequencies the internal space charges can no longer react (this is because the space charges cannot keep up with fast switching polarity of the electric field, i.e., the electric field switches rapidly and the space charges cannot react to it thereby negating its effect) and therefore, has no effect on the net polarization. Furthermore, the orientation of the polar groups is relatively slow and once the frequency increases, tends to lag behind. A further increase in frequency manifests in the inactivity of the dipole contributions towards polarization, i.e., only random orientations remain, and these do not contribute to the resultant polarization. Of the total polarization, now only the atomic and electronic polarization remain. At even further frequencies the stretching and bending of the bonds start to slacken, thereby negating the effect of the ionic polarization. Finally, at much higher frequencies ~10^15^ Hz the distortion of electronic clouds from the nuclei begins to lag behind.

**Figure 3:**
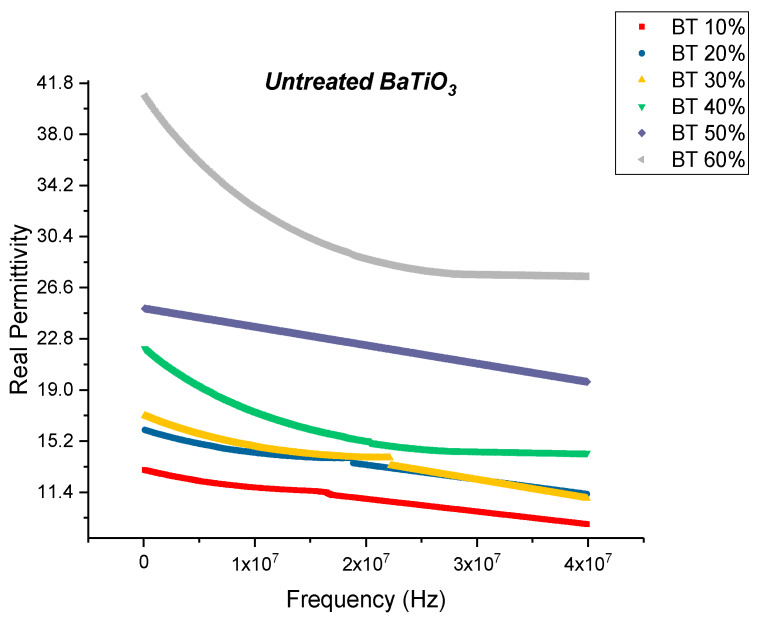
The real permittivity of the BaTiO_3_-epoxy composite that were fabricated using untreated BaTiO_3_ nanoparticles is plotted as a function of frequency where the volume fraction of BaTiO_3_ is varied from 10% to 60%. This value at 20 MHz attains a maximum of 28.70 at 60% volume fraction of BaTiO_3_.

The real permittivity value at 20 MHz attains a maximum value of 28.70 for the untreated BaTiO_3_/epoxy composite where the volume fraction of BaTiO_3_ was 60% as shown in Figure 3. The dissipation factor is defined as the ratio of imaginary part of permittivity i.e., dielectric loss with the real part of permittivity i.e., relative permittivity or dielectric constant. Therefore, the dielectric loss can be calculated my multiplying the dissipation factor and the relative permittivity. The maximum dissipation factor at 20 MHz was recorded to be 0.3836 at a volume fraction of 60% of BaTiO which is comparable to that reported by Dalle Vacche et al. (~0.4) [51]. It is important to note that the dissipation factor increases quite significantly at 60% of BaTiO_3_, as can be seen in Figure 5 and this could be attributed to the increase in agglomerations due to lack of wettability of the powders, and hence causing inhomogeneous dispersion of particles. This creates more air voids within the sample, increasing its porosity and making it difficult to pole, as the air voids are prone to increase in electric conduction.

**Figure 4:**
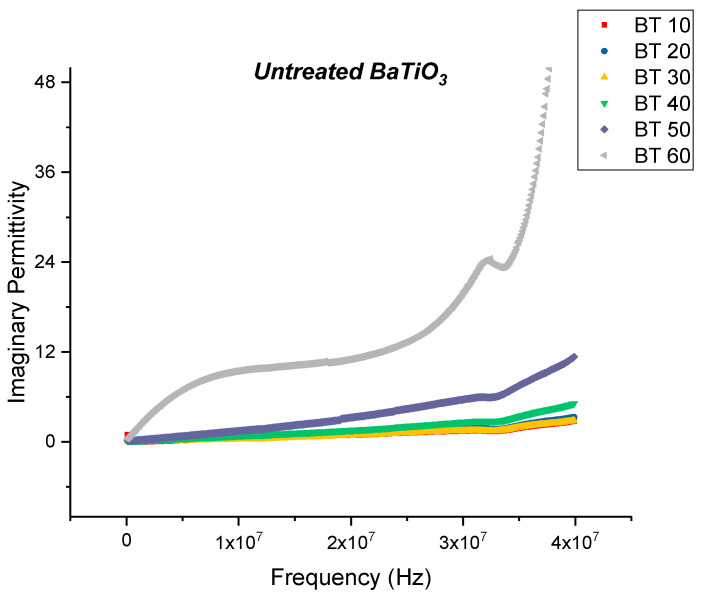
The dielectric loss of the BaTiO_3_-epoxy composite that were fabricated using untreated BaTiO_3_ nanoparticles is plotted as a function of frequency where the volume fraction of BaTiO_3_ is varied from 0.1 to 0.6. The maximum dielectric loss at 20 MHz was recorded to be 11.06 at a volume fraction of 0.6 of BaTiO_3_.

**Figure 5:**
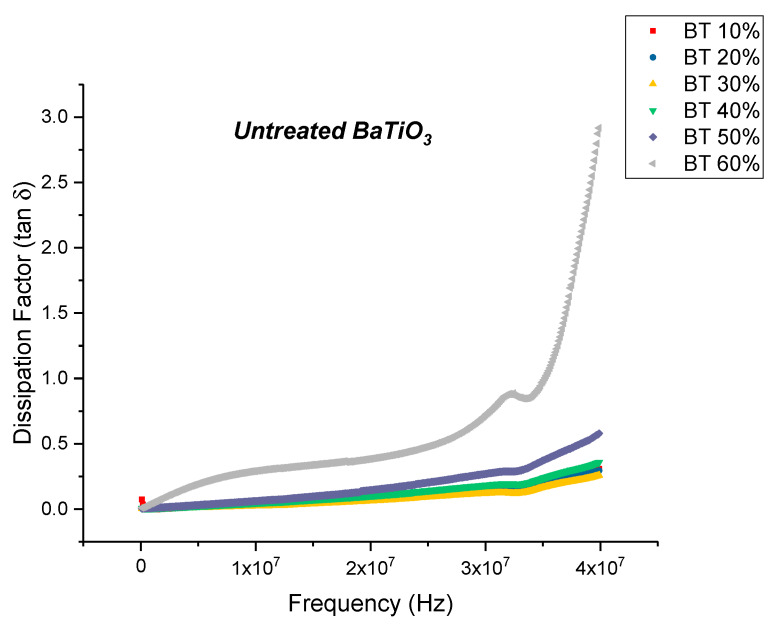
The dissipation factor of the BaTiO_3_-epoxy composite that were fabricated using untreated BaTiO_3_ nanoparticles is plotted as a function of frequency where the volume fraction of BaTiO_3_ is varied from 0.1 to 0.6. The maximum dissipation factor at 20 MHz was recorded to be 0.3836 at a volume fraction of 0.6 of BaTiO_3_.

The dielectric loss and dissipation factor for the composite that were prepared using untreated BaTiO_3_ nanoparticles are plotted as a function of increasing frequency as shown in Figure 4 and Figure 5, respectively, where the volume fraction of BaTiO_3_ was varied from 0.1 to 0.6. The maximum dielectric loss at 20 MHz was recorded to be 11.06 at a volume fraction of 0.6 of BaTiO_3_.

**Figure 6:**
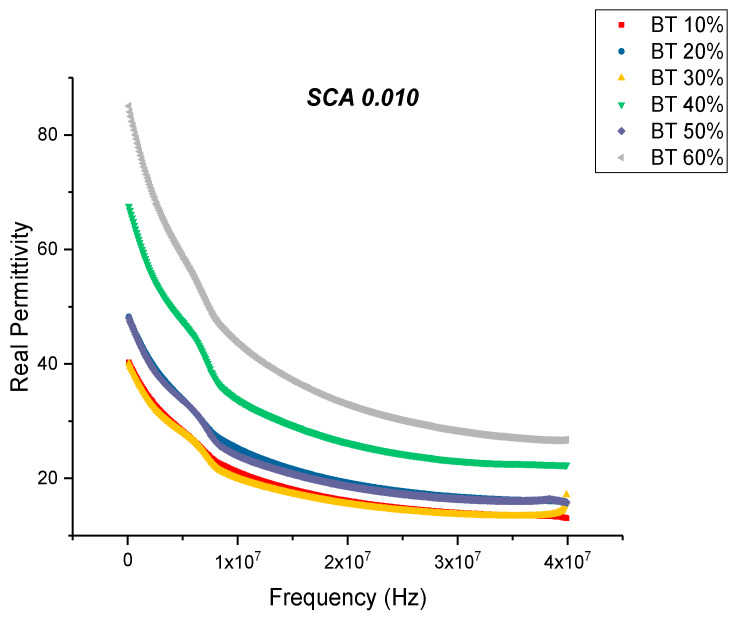
The real permittivity of the BaTiO_3_-epoxy composite that were fabricated using BaTiO_3_ nanoparticles which were surface modified using 0.010 SCA is plotted as a function of frequency where the volume fraction of BaTiO_3_ is varied from 0.1 to 0.6.

The permittivity of the 0.01 surface treated samples also show an increase in value with increasing volume fraction of BaTiO_3_ as shown in Figure 6. The maximum value of real permittivity at 20 MHz was recorded to be 32.80 at 60% volume fraction of BaTiO_3_. This is approximately 14.28% increase compared to the untreated samples. It was also found that at even high concentrations of BaTiO_3_ the composite did not crack or delaminate due to the improved dispersion, this observation has been made by several researchers [13,21,24,47,50,77]. With the addition of the coupling agent, a more homogeneous and uniform dispersion can be obtained. The enhanced dispensability of the nanoparticles can mainly be attributed to the substitution of the hydroxyl groups, which has a dual effect, (i) lowering interfacial tension between the separated phases and (ii) preventing coalescence of the nanoparticles during processing [47].

**Figure 7:**
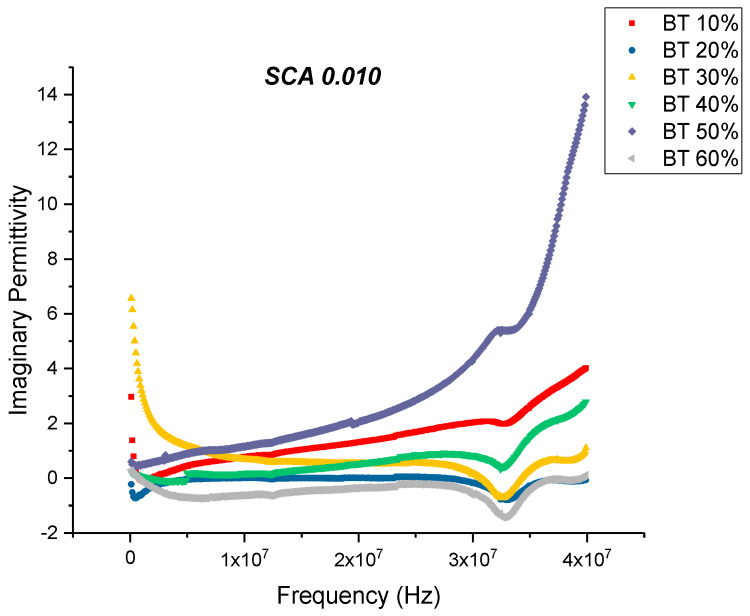
The dielectric loss of the BaTiO_3_-epoxy composite that were fabricated using BaTiO_3_ nanoparticles which were surface modified using 0.010 SCA is plotted as a function of frequency where the volume fraction of BaTiO_3_ is varied from 0.1 to 0.6. The maximum value is 2.05 for BaTiO_3_ at 0.5 volume fraction. The imaginary permittivity increases with increasing volume fraction of BaTiO_3_ nanoparticles and increases with increasing frequency. With increasing frequency, there are fewer contributing factors to the real permittivity do the delayed response of these orientations.

**Figure 8:**
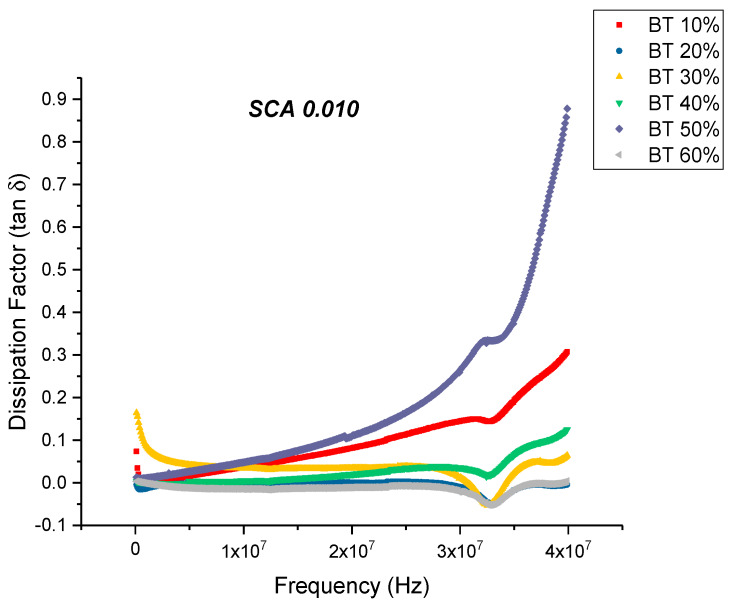
The dissipation factor of the BaTiO_3_-epoxy composite that were fabricated using BaTiO_3_ nanoparticles which were surface modified using 0.010 SCA is plotted as a function of frequency where the volume fraction of BaTiO_3_ is varied from 0.1 to 0.6. The maximum value is 0.1106 for BaTiO_3_ at 0.5 volume fraction.

From Figure 7 and Figure 8, one can see that the dissipation factor and dielectric loss for the SCA 0.01 samples behaves similar to that of the untreated ones, wherein the dissipation factor begins to increase with increasing frequency. This is synonymous with the decrease in permittivity over the same frequency range. A relaxation process of the polymer matrix leads to a decrease in the permittivity values from low frequency to high frequency values. The dissipation factor at 20 MHz attains a maximum value of 0.1106, which is significantly lower compared to the untreated samples; 0.3836. For composite systems, the dielectric loss factor might originate from the contributions of dipole orientation, conduction loss and interfacial polarization [47,78]. The loss factor could be expressed as a sum of three distinct effects [79] namely, conduction loss contribution, interfacial polarization and the dipole orientation or Debye loss factor. Surface treatment of the BaTiO_3_ nanoparticles minimizes the conduction losses due to substitution of the hydroxyl groups, which would have been present at the BaTiO_3_-epoxy interface.

**Figure 9:**
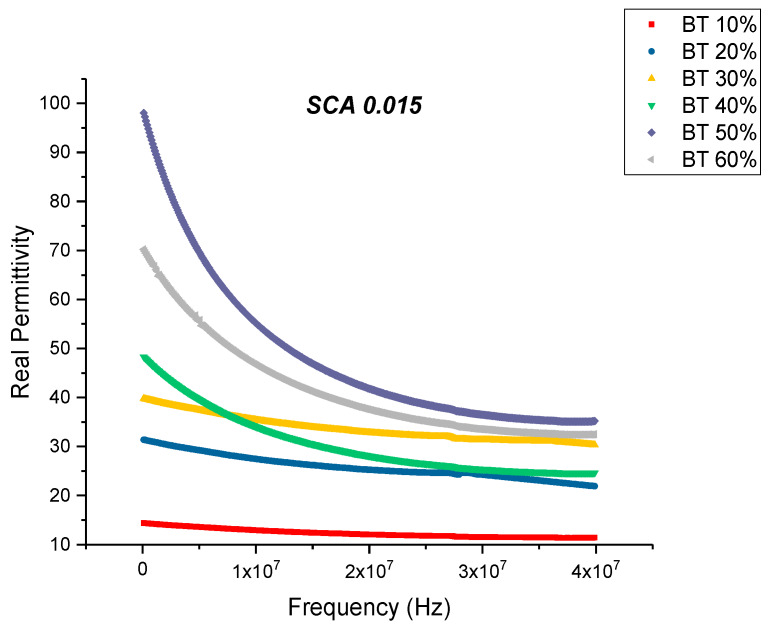
The real permittivity of the BaTiO_3_-epoxy composite that were fabricated using BaTiO_3_ nanoparticles which were surface modified using 0.015 SCA is plotted as a function of frequency where the volume fraction of BaTiO_3_ is varied from 0.1 to 0.6.

For the samples that were surface treated using 0.015 concentration of silane coupling agent, the relative permittivity shows an increasing tendency with the loading levels of the nanoparticles as shown in Figure 9. The maximum permittivity values at 20 MHz was found to be 41.76 at 50% volume fraction of BaTiO_3_. This value is a 27% increase compared to the permittivity of the SCA 0.015 sample. The loss factor begins to increase with increasing frequency and can be attributed to the decrease in reorientation of dipoles and polar entities, it may also originate from the contributions of conduction loss and interfacial polarization [47,78].

**Figure 10:**
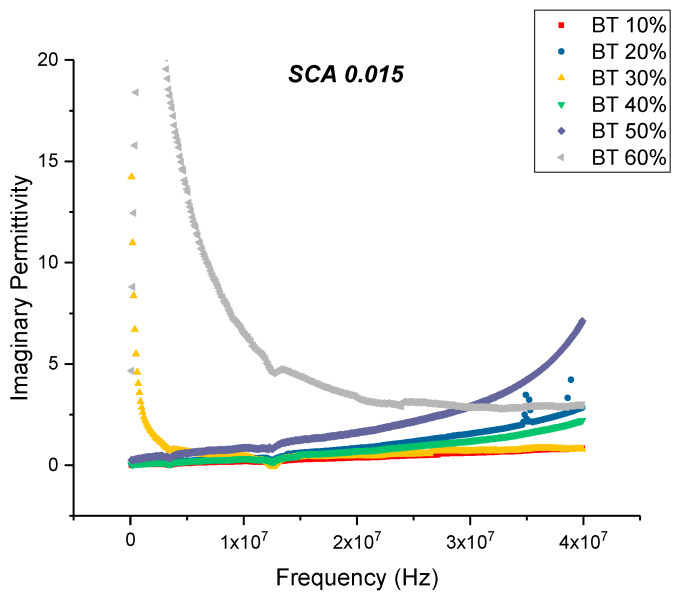
The dielectric loss of the BaTiO_3_-epoxy composite that were fabricated using BaTiO_3_ nanoparticles which were surface modified using 0.015 SCA is plotted as a function of frequency where the volume fraction of BaTiO_3_ is varied from 0.1 to 0.6. The maximum value is 1.60 for BaTiO_3_ at 0.6 volume fraction.

**Figure 11:**
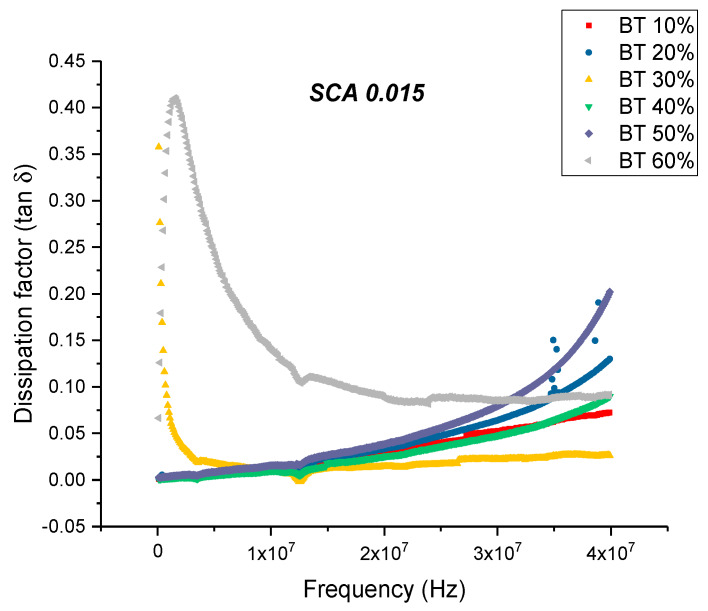
The dissipation factor of the BaTiO_3_-epoxy composite that were fabricated using BaTiO_3_ nanoparticles which were surface modified using 0.015 SCA is plotted as a function of frequency where the volume fraction of BaTiO_3_ is varied from 0.1 to 0.6. The maximum value is 0.087 for BaTiO_3_ at 0.6 volume fraction. The imaginary permittivity increases with the increasing volume fraction of BaTiO_3_ nanoparticles and increases with increasing frequency. With increasing frequency, there are fewer contributing factors to the real permittivity do the delayed response of these orientations.

The maximum dielectric loss and dissipation factor for BaTiO_3_/epoxy composites prepared using SCA 0.015 occurs at 60% volume fraction of BaTiO_3_ as shown in Figure 10 and Figure 11. The trend observed for the dissipation factor shows an increase in value with increasing volume fraction of BaTiO_3_. This increase in dissipation factor can be attributed to the increased conduction losses associated with increasing amount of BaTiO_3_.

This is an almost 21% decrease in dissipation compared to the maximum value exhibited by the composites prepared using SCA 0.01. The decrease in dissipation factor, as previously mentioned, could be attributed to better interfacial bonding between the BaTiO_3_ nanoparticles and the epoxy matrix because the coupling agent acts as a molecular bridge between the two, and therefore, contributes to lower conduction losses [80,81].

**Figure 12:**
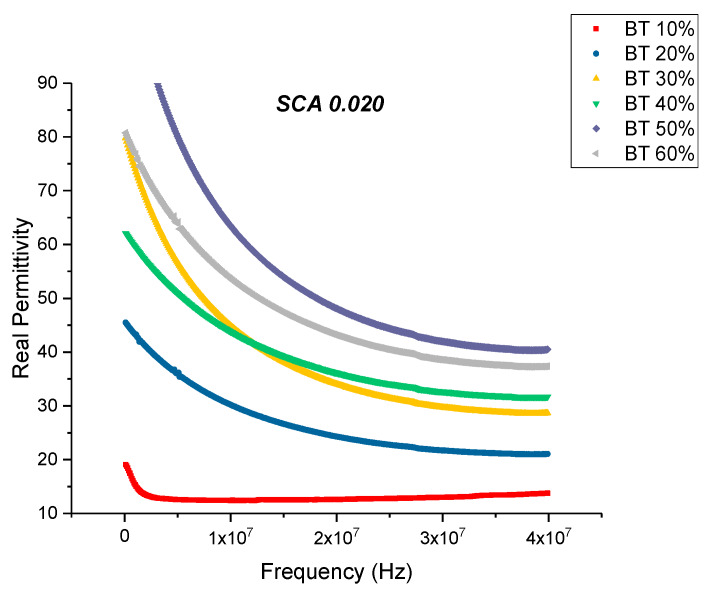
The real permittivity of the BaTiO_3_-epoxy composite that were fabricated using BaTiO_3_ nanoparticles which were surface modified using 0.020 SCA is plotted as a function of frequency where the volume fraction of BaTiO_3_ is varied from 0.1 to 0.6.

**Figure 13:**
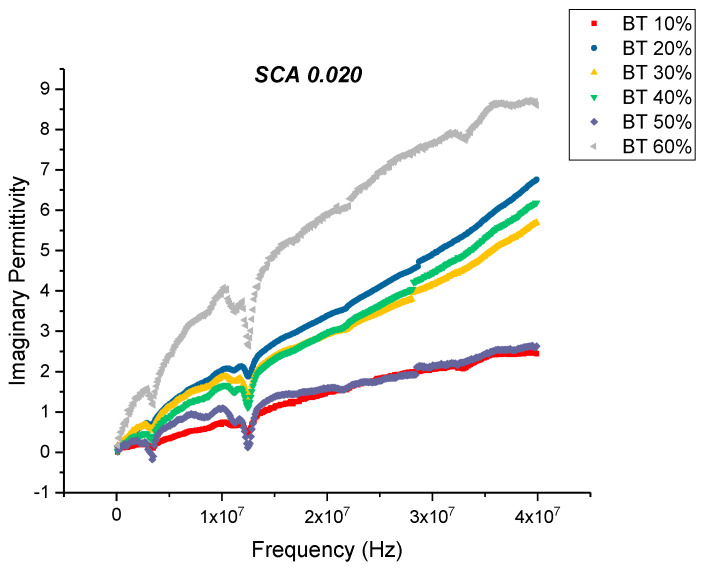
The dielectric loss of the BaTiO_3_-epoxy composite that were fabricated using BaTiO_3_ nanoparticles which were surface modified using 0.020 SCA is plotted as a function of frequency where the volume fraction of BaTiO_3_ is varied from 0.1 to 0.6. The maximum value is 5.89 for BaTiO_3_ at 0.6 volume fraction.

**Figure 14:**
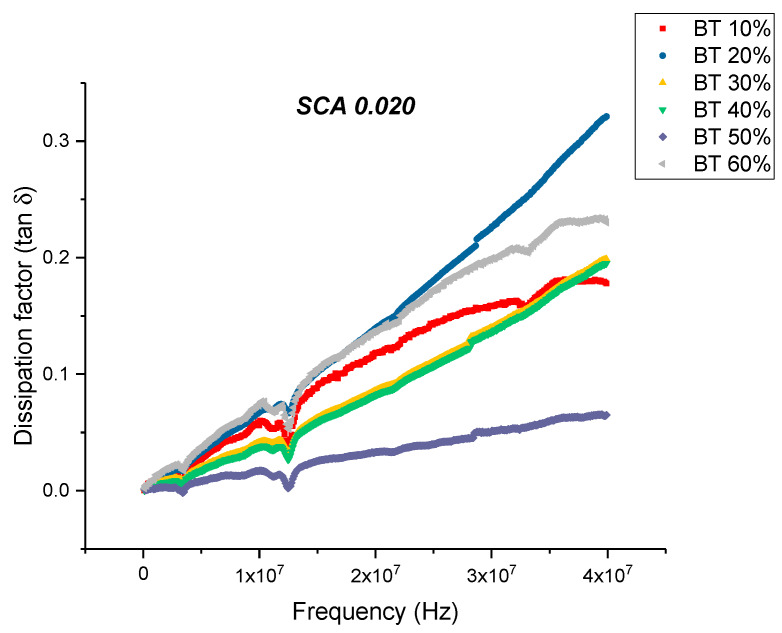
The dissipation factor of the BaTiO_3_-epoxy composite that were fabricated using BaTiO_3_ nanoparticles which were surface modified using 0.020 SCA is plotted as a function of frequency where the volume fraction of BaTiO_3_ is varied from 0.1 to 0.6. The maximum value is 0.136 for BaTiO_3_ at 0.6 volume fraction.

There is a noticeable decrease in the real part permittivity as a function of frequency as shown in Figure 12, for BT with a 0.020 concentration of silane. The dielectric relaxations depicted in Figure 12 reflects polarization mode changes. There is a further decrease in dielectric loss and dissipation factor for the composite that were surface modified using SCA 0.020 as shown in Figure 13 and Figure 14. For these composites prepared using 0.5 volume fraction of BaTiO_3_ the dissipation factor exhibits a value of 0.033 as shown in Figure 14.

**Figure 15:**
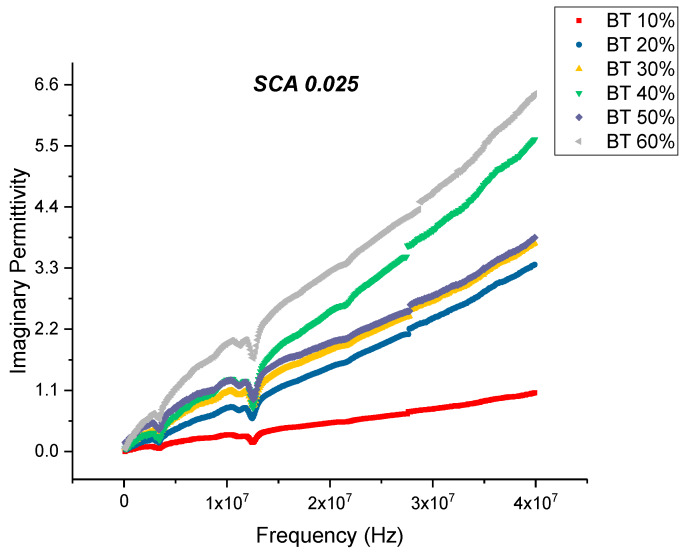
The dissipation factor of the BaTiO_3_-epoxy composite that were fabricated using BaTiO_3_ nanoparticles which were surface modified using 0.025 SCA is plotted as a function of frequency where the volume fraction of BaTiO_3_ is varied from 0.1 to 0.6. The maximum value is 3.26 for BaTiO_3_ at 0.6 volume fraction.

**Figure 16:**
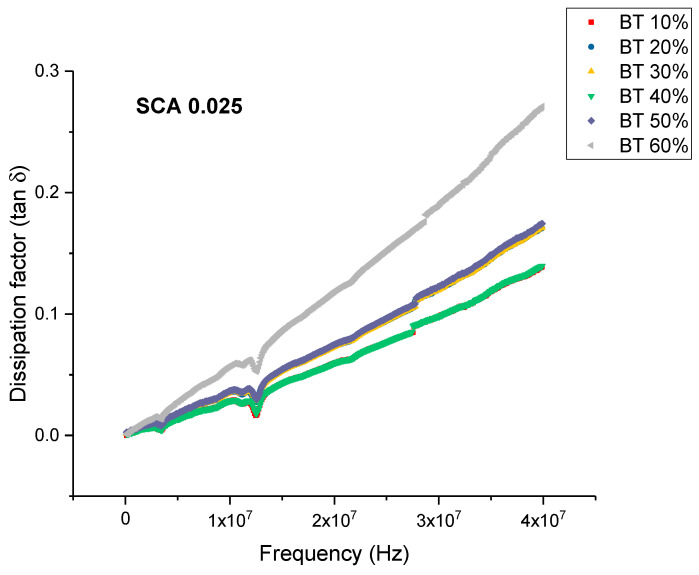
The dissipation factor of the BaTiO_3_-epoxy composite that were fabricated using BaTiO_3_ nanoparticles which were surface modified using 0.025 SCA is plotted as a function of frequency where the volume fraction of BaTiO_3_ is varied from 0.1 to 0.6. The maximum value is 0.119 for BaTiO_3_ at 0.6 volume fraction.

**Figure 17:**
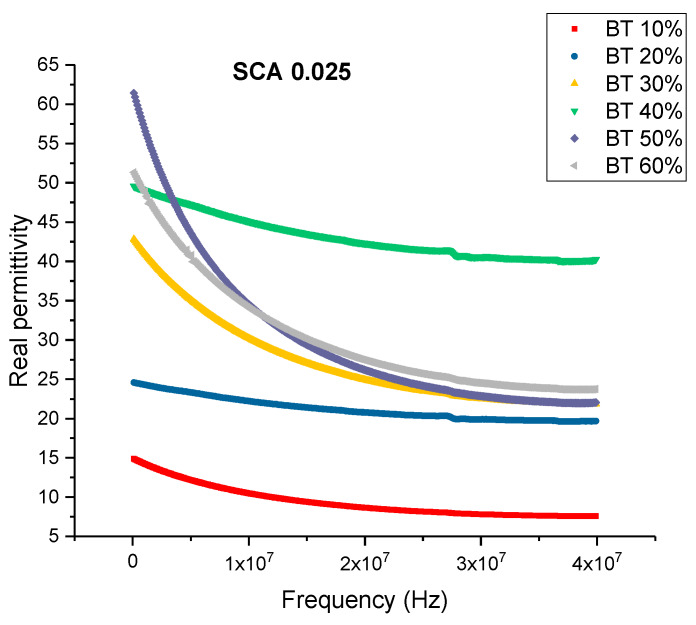
The real permittivity of the BaTiO_3_-epoxy composite that were fabricated using BaTiO_3_ nanoparticles which were surface modified using 0.025 SCA is plotted as a function of frequency where the volume fraction of BaTiO_3_ is varied from 0.1 to 0.6.

When the concentration of coupling agent is increased to 0.025 and for higher BaTiO_3_ volume fractions, the dissipation factor began to increase again slowly as shown in Figure 15. From Figure 16, the maximum dissipation factor was found to be 0.119 at 0.6 volume fraction of BaTiO_3_. The concentration of silane coupling agent does not achieve as desirable of permittivity results for a concentration of 0.025 in comparison with the other concentrations of silane coupling agent as shown in Figure 17, which demonstrates further the importance of concentration of silane agent.

**Figure 18:**
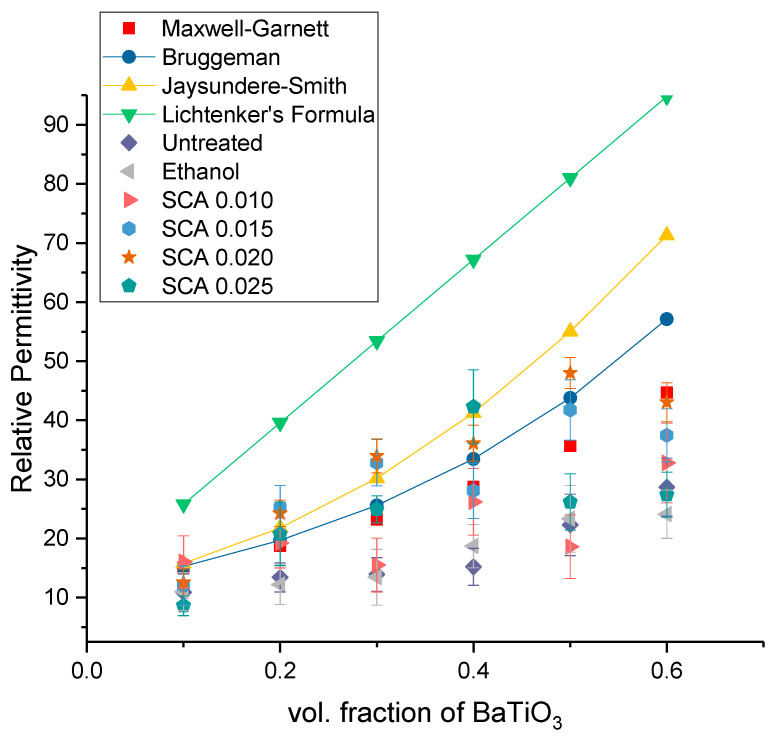
Comparison of the relative permittivity of the theoretical models with different experimental results, where the readings were taken at 20 MHz for the composites that were prepared using 0.5 volume fraction of BaTiO_3_.

A comparison of the experimental relative permittivity values and theoretical models is plotted in Figure 18. The mathematical models used were the Maxwell-Garnett equation, Bruggeman self-consistent effective medium approximation, the Jaysundere-Smith equation and Lichtenker rule. Table 3 lists the values of the values obtained from the mathematical models and the experimental values obtained at 20 MHz.

A comparison of our work with the dielectric constants of other notable workers is presented in in Table 4, where our work demonstrates that optimization of surface treatment leads to enhanced properties by a factor of ~1.5 in comparison to our counterparts that employ non-piezoelectric matrix materials. It also demonstrates that optimization of surface treatment can lead to permittivity values that are very close to those that incorporate a piezoelectric-active matrix.

#### Conductivity (Untreated BTO and BTO-Silane Coupling Agent)

**Figure 19:**
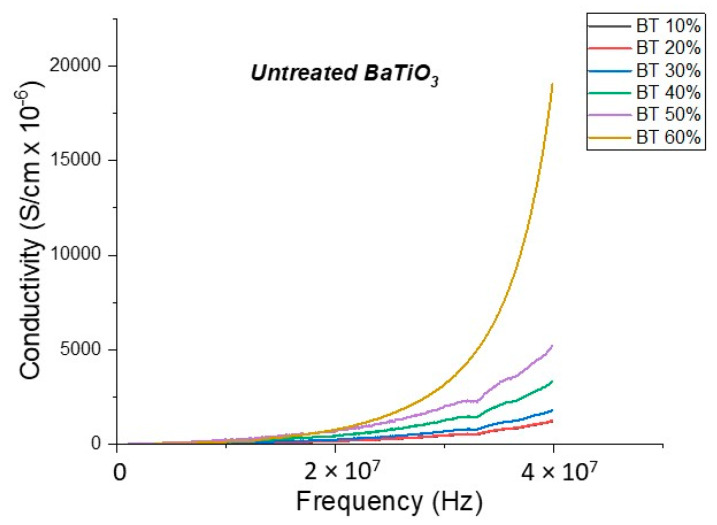
The conductivity of the BaTiO_3_-epoxy composite that were BaTiO_3_ nanoparticles which were not surface treated (pristine) is plotted as a function of frequency. The maximum value of conductivity at 20 MHz is 7.84 × 10^−4^ S/m and occurs at BT 0.6.

**Figure 20:**
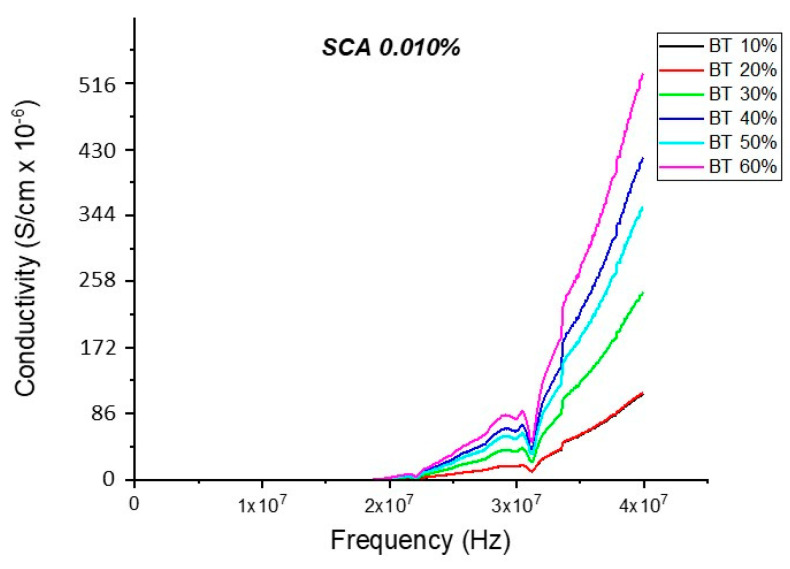
The conductivity of the BaTiO_3_-epoxy composite that were fabricated using BaTiO_3_ nanoparticles which were surface modified using 0.010 SCA is plotted as a function of frequency. The maximum value of conductivity at 20 MHz is 4.63 × 10^−4^ S/m and occurs at BT 0.6.

**Figure 21:**
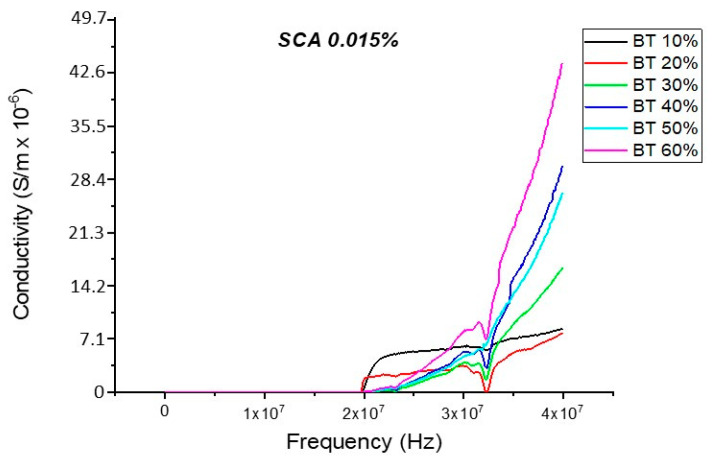
The conductivity of the BaTiO_3_-epoxy composite that were fabricated using BaTiO_3_ nanoparticles which were surface modified using 0.015 SCA is plotted as a function of frequency. The maximum value of conductivity at 20 MHz is 4.36 × 10^−4^ S/m and occurs at BT 0.6.

**Figure 22:**
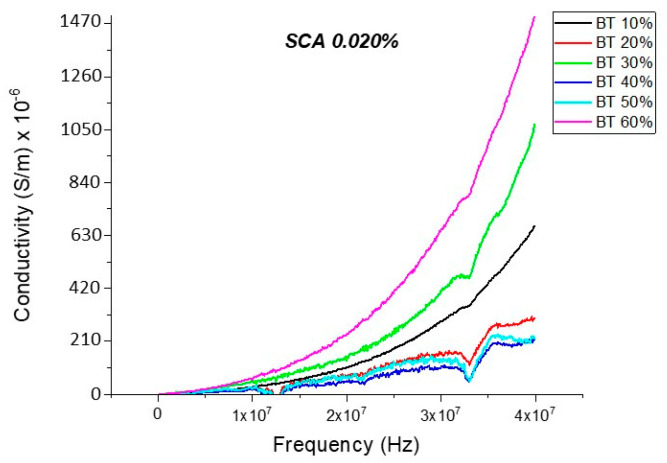
The conductivity of the BaTiO_3_-epoxy composite that were fabricated using BaTiO_3_ nanoparticles which were surface modified using 0.020 SCA is plotted as a function of frequency. The maximum value of conductivity at 20 MHz is 2.38 × 10^−4^ S/m and occurs at BT 0.6.

**Figure 23:**
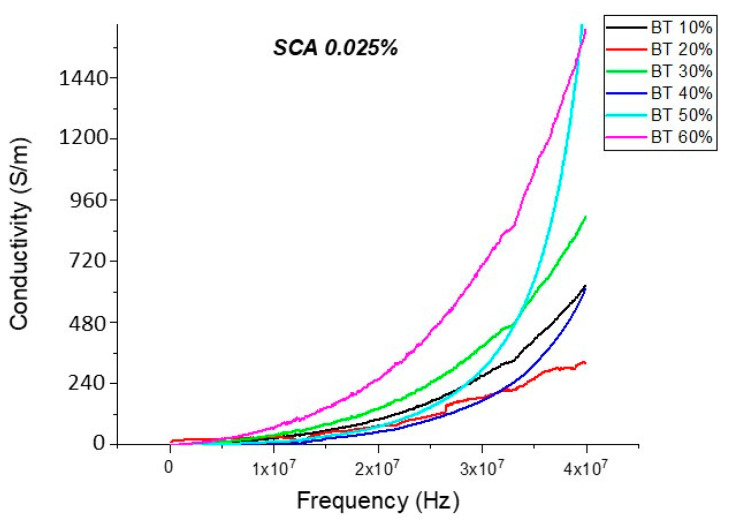
The conductivity of the BaTiO_3_-epoxy composite that were fabricated using BaTiO_3_ nanoparticles which were surface modified using 0.025 SCA is plotted as a function of frequency. The maximum value of conductivity at 20 MHz is 2.58 × 10^−4^ S/m and occurs at BT 0.6.

The conductivity measurements, as expected, showed a similar trend to that of the resistivity observations as shown in Figure 19, Figure 20, Figure 21, Figure 22 and Figure 23. For the composites prepared with 0.5 volume fraction of BaTiO_3_ and surface modified using SCA 0.015, SCA 0.0 and SCA 0.025 the conductivity values were 2.61 × 10^−4^ S/m, 7.02 × 10^−5^ S/m and 7.3 × 10^−5^ S/m, respectively.

**Figure 24:**
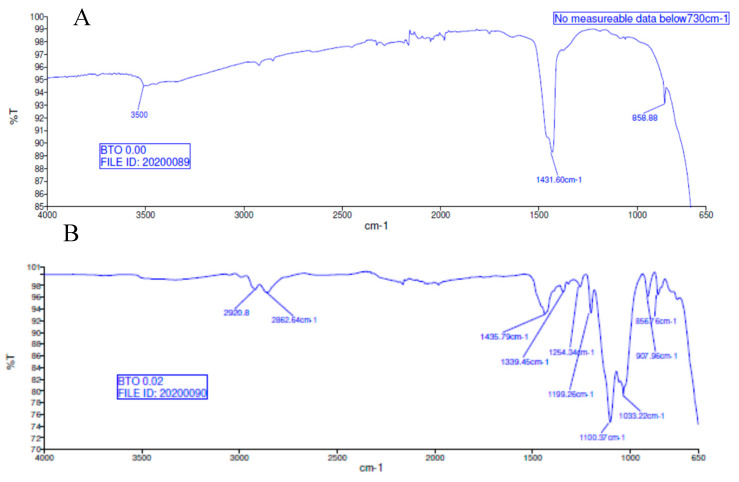
FTIR spectra of (**A**) BTO and (**B**) surface modified BTO particles.

There is a decrease in conductivity of the SCA 0.015 and SCA 0.020 surface treated samples by ~73% 20 MHz in 50% BaTiO_3_ samples as shown in Figure 21, Figure 22 and Figure 23. Moreover, the conductivity of the composites decreases as a function of silane coupling agent volume fraction. The pristine or untreated BaTiO_3_ tends to adsorb water onto its surface due to its hydrophilic nature [13,14]. The silane coupling surface modification leads to the substitution of the hydroxyl (–OH–) groups on the surface of the BaTiO_3_ filler that has a modified surface chemistry of (O–Si–) as shown in Figure 24. As indicated in the FTIR plot in Figure 24, as the concentration of coupling agent increases, more reactions may occur, which lead to the reduction in electrical conductivity [21,77].

**Figure 25:**
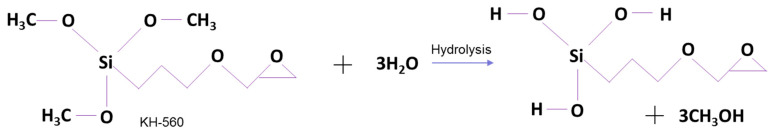
The alkoxide groups (Si–OCH_3_) of KH-560 are hydrolyzed by water thereby leading to the formation of Si–OH groups and methanol.

**Figure 26:**
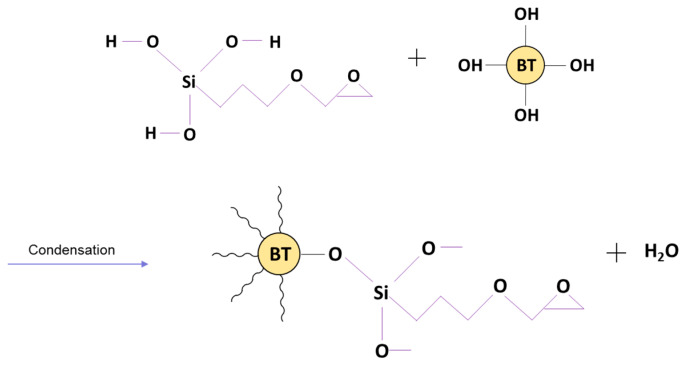
A condensation reaction occurs between the newly formed Si–OH groups of the coupling agent and the hydroxyl groups adsorbed on the surface of the BaTiO_3_ nanoparticle.

In Figure 24, Fourier-transform infrared spectroscopy (FTIR) of the BTO powder (A) and surface treated BTO powder (B) are presented. The reaction mechanism leading to enhance dielectric permittivity is due to the coupling agent and modified surface of the BTO whose interfacial bond with the epoxy matrix is enhanced by way of the addition of the silane coupling agent as indicated in Figure 25. The bands at 1435–1390 cm^−1^ represent BTO, while the broad peak centered at 3500 cm^−1^ is attributed to hydroxyl groups on the BTO nanoparticles which are present in the as-made BTO. The bands between 850–900 represent the Si–O (Si–OH) stretching vibration band groups, while the range from 2850–2950 cm^−1^ represents the CH and CH_2_ stretching aliphatic group, detailed in Table 5. The silane coupling agent, KH-560, contains an organic functional group that resembles an epoxy chain and three alkoxide groups viz. (Si–OCH_3_). During the surface modification procedure, which involves mixing KH-560, ethanol and BaTiO_3_, the alkoxide groups are hydrolyzed by water generating Si–OH groups and alcohols, as can be seen in Figure 25 and Figure 26. The water that reacts with the alkoxide groups can come from two sources namely, (i) the solvent, in this case ethanol (95/5; ethanol/water) or (ii) water adsorbed on particle surface when an anhydrous solvent is used.

**Figure 27:**
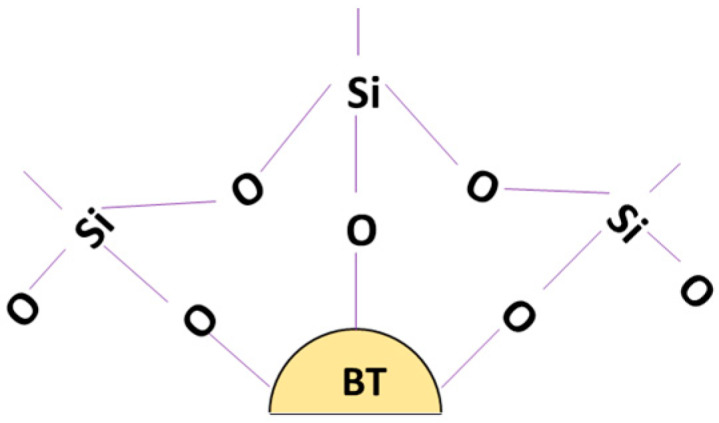
The surface of the BaTiO_3_ nanoparticle, which previously contained hydroxyl groups now is replaced by a coupling agent which can bond easily with epoxy resin.

The hydrolysis reaction is followed by a condensation reaction between the newly formed Si–OH groups of the coupling agent and the hydroxyl groups on the surface of the BaTiO_3_ nanoparticle, as shown in Figure 26. This causes BaTiO_3_ to rid itself of the hydroxyl group on its surface with a coupling agent that can bond easily with epoxy as can be seen in Figure 27. The condensation reaction leads to the formation of water and BaTiO_3_ particles with a modified surface chemistry. This reaction makes the BT nanoparticle surface more compatible with the epoxy matrix.

If multiple such reactions occur, then more hydroxyl groups are available to react with the coupling agent, leading to the formation a modified BaTiO_3_ particle as shown in Figure 27.

The results above indicate that for a single particle surface the hydroxyl (–OH–) groups have been substituted with that of the coupling agent after the condensation of the silanol groups. This implies that the BaTiO_3_ can bond easily with the epoxy (Bisphenol A diglycidyl ether) matrix due to the ‘glycidyl’ functional group of the coupling agent (ɣ-glycidyloxytrimethoxysilane).

## 4. Conclusions

Silicon molecules that were adsorbed onto the surface of the BaTiO_3_ nanoparticle were observed using the EDS micrograph images, which also showed the reduction in the aggregate size, which led to better particle distribution. The highest value of permittivity (~48.03) and the lowest value of loss (~0.136) were observed for the samples that were fabricated using 0.5 volume fraction of BaTiO_3_ and that were surface treated with 0.02 volume fraction of silane coupling agent. The improved adhesion between the polymer-ceramic interface led to higher interfacial polarization within the composite material and resulted in increased permittivity values. Due to the insulating adsorption layer of the organofunctional silane the localized conductivity at the interface was reduced leading to enhanced permittivity values.

## Figures and Tables

**Table 1:** Physical properties of BaTiO_3_.

Barium Titanate Nano-Powder	
**Property**	
Density (g/cm^3^)	6.08
Mean Diameter *	~100 nm
Curie Point (°C)	130 °C
Dielectric constant	150 [54]
*d_33_* (pC/N)	~85.6 (crystal)/~191 (ceramic)

* As received from manufacturer.

**Table 2:** Physical, dielectric and piezoelectric properties of DGEBA epoxy.

Property	
Dielectric Constant	2.9–3.0 *
Dielectric dissipation	~0.02–0.04
Electromechanical coupling factor, *k_33_*	-
Piezoelectric strain coefficient, *d_33_*	-
Density	1.16 g/cm^3^
Electrical Resistivity	0.15 @ 1 kHz

* Experimental values.

**Table 3:** Comparison of the relative permittivity of the theoretical models with different experimental results, where the readings were taken at 20 MHz for the composites that were prepared using 0.5 volume fraction of BaTiO_3_.

Symbol	Legend	Relative Permittivity	Standard Deviation
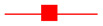	Maxwell-Garnett Equation	35.65	-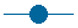	Bruggeman Self-Consistent Effective Medium Approximation	43.80	-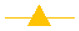	Jaysundere-Smith Equation	55.02	-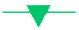	Lichtenker’s Formula	81	-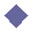	Untreated BaTiO_3_	22.31	5.2
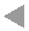	Ethanol Surface Treated	23.36	5.64
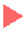	Silane Coupling Agent—0.010	18.62	5.37
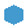	Silane Coupling Agent—0.015	41.76	5.11
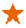	Silane Coupling Agent—0.020	48.03	5.63
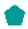	Silane Coupling Agent—0.025	26.20	4.78

**Table 4:** Comparison of this work effort with other notable researchers who have fabricated state of the art materials.

Type of Composite	Surface Modification	Dielectric Permittivity
BaTiO_3_—epoxy composite: thin film capacitors [82]	N-phenyl aminopropyltrimethoxysilane	40 @ 1 kHz
BaTiO_3_—epoxy composite [83,84]	3-aminoprpoyltriethoxysilane (KH-550)	vol. fraction < 70%. ~35 @ 10^5^ Hz
BaTiO_3_/epoxy composites [85,86]	3-glycidoxypropyltrimethoxysilane	~28 @10^5^ Hz
BaTiO_3_ /(EVM) copolymer elastomer [35,85]	γ-aminopropyl triethoxysilane (Silquest A-1100)	Increasing permittivity with BaTiO_3_ loading. ~25 @ 70 wt.% of BaTiO_3_
BTresin/BaTiO_3_ [85,86]	3-glycidoxypropyl trimethoxysilane (KH-560)	Permittivity increased with loading. ~5 at 0%–~25 at 70%
BaTiO_3_/epoxy: Integral thin film capacitors [35,85]	N-phenyl aminopropyltrimethoxysilane, glycidoxy, mercapto, cyclohexyl	~40 @ 10^5^ Hz
BaTiO_3_-epoxy composites [56]	Glycidoxymethoxysilane Z-6040	Increases with particles that were surface modified ~40 at 100 Hz
DGEBA-forsterite composites [85]	Aminopropyltriethoxy silane (APTS)	Dielectric permittivity increased from 3.7 to 3.9 after surface treating.
BaTiO_3_-P(VDF-TrFE) [57]	3-aminopropyltriethoxysilane (APTS)	Dielectric permittivity improved—(i) 100 to 113, (ii) 80−100 and (ii) 75−85
BaTiO_3_-DEGBA epoxy (this work)	γ–glycidyloxypropyltrimethoxysilane (KH-560)	76 @ 10 kHz (0.20 SCA) 97 @ 100 Hz (0.150 SCA) 88 @ 1 kHz (0.20 SCA)

**Table 5:** Wavelength (cm^−1^) and bond type found in silane films.

Wavelength (cm^−1^)	Bond Type
700–800	C–H (Si–CH_2_–CH_2_–Si) stretching vibrations
850–900	Si–O (Si–OH) stretching vibrations
900–960	Si–O–C_2_H_5_ vibrations
1000–1250	Si–O (from Si–O–Si bonds) vibrations
1300–1400	CH_2_ and CH_3_ bending vibrations
3200–3700	OH (from Si–OH group) stretching vibrations
